# Re-emergence of Rabies in the Guangxi Province of Southern China

**DOI:** 10.1371/journal.pntd.0003114

**Published:** 2014-10-02

**Authors:** Hai-Bo Tang, Yan Pan, Xian-Kai Wei, Zhuan-Ling Lu, Wu Lu, Jian Yang, Xiao-Xia He, Lin-Juan Xie, Lan Zeng, Lie-Feng Zheng, Yi Xiong, Nobuyuki Minamoto, Ting Rong Luo

**Affiliations:** 1 State Key Laboratory for Conservation and Utilization of Subtropical Agro-bioresources, Guangxi University, Guangxi, China; 2 College of Animal Sciences and Veterinary Medicine, Guangxi University, Guangxi, China; 3 Guangxi Center for Animal Disease Control and Prevention, Guangxi, China; The Global Alliance for Rabies Control, United States of America

## Abstract

**Background:**

Human rabies cases in the Guangxi province of China decreased from 839 in 1982 to 24 in 1995, but subsequently underwent a sharp increase, and has since maintained a high level.

**Methodology/Principal Findings:**

3,040 brain samples from normal dogs and cats were collected from 14 districts of Guangxi and assessed by RT-PCR. The brain samples showed an average rabies virus (RV) positivity rate of 3.26%, but reached 4.71% for the period Apr 2002 to Dec 2003. A total of 30 isolates were obtained from normal dogs and 28 isolates from rabid animals by the mouse inoculation test (MIT). Six representative group I and II RV isolates showed an LD_50_ of 10^−5.35^/ml to 10^−6.19^/ml. The reactivity of monoclonal antibodies (MAbs) to group I and II RV isolates from the Guangxi major epidemic showed that eight anti-G MAbs showed strong reactivity with isolates of group I and II with titers of ≥10,000; however, the MAbs 9-6, 13-3 and 12-14 showed lower reactivity. Phylogenetic analysis based on the G gene demonstrated that the Guangxi RV isolates have similar topologies with strong bootstrap values and are closely bonded. Alignment of deduced amino acids revealed that the mature G protein has four substitutions A96S, L132F, N436S, and A447I specific to group I, and 13 substitutions T90M, Y168C, S204G, T249I, P253S, S289T, V332I, Q382H, V427I, L474P, R463K Q486H, and T487N specific to group II, coinciding with the phylogenetic analysis of the isolates.

**Conclusions:**

Re-emergence of human rabies has mainly occurred in rural areas of Guangxi since 1996. The human rabies incidence rate increased is related with RV positive rate of normal dogs. The Guangxi isolates tested showed a similar pathogenicity and antigenicity. The results of phylogenetic analysis coincide with that of alignment of deduced amino acids.

## Author Summary

Rabies is a worldwide zoonosis disease and is of considerable public health threat and hazard. The Guangxi province of southern China is a severe rabies epidemic region. Human rabies cases decreased from 839 in 1982 to 24 in 1995 in Guangxi as a result of a dog vaccination campaign. However, the number subsequently underwent a sharp increase, and has since maintained a high level. This study reports the systematic surveillance of rabies in Guangxi over the 30-year period from 1982 to 2012. The data revealed that a re-emergence of human rabies has occurred mainly in rural areas of Guangxi since 1996. Human rabies incidence rate increased follows increased instances of RV positive normal dogs. To further understand this re-emergence of rabies, the biological properties of the rabies virus (RV), including the RV-positive rate of normal dogs, pathogenicity, antigenicity and evolution, have been evaluated. The Guangxi isolates all showed similar pathogenicity and antigenicity. These isolates also exhibited similar topologies with strong bootstrap values in the two groups and were closely bonded. Thus these findings will be helpful to understanding the epidemiological situation for rabies in Guangxi.

## Introduction

Rabies is a fatal enzootic viral infection of the central nervous system. The disease is widespread throughout the world, and is a serious public health problem in developing countries. The WHO reported that human mortality from endemic canine rabies is estimated to be 55000 deaths per year in Asia and Africa, with 56% of these deaths occurring in Asia. The majority (84%) of these deaths occur in rural areas [1]. Dogs are the principal host of the rabies virus and play a primary role in rabies transmission in Asia. More recently, several reports on the molecular epidemiology of rabies have been published from Asian countries, such as Thailand [2], Indonesia [3], South Korea [4], and China [5]–[6].

The rabies virus (RV) is a member of the Lyssavirus genus and is distributed in a wide range of host species. RV has been extensively studied because of its significant impact on public health, especially considering that it is fatal in people. In China, epidemiological surveillance has shown a re-emergence of human rabies since 1995. Using molecular characterization based on the genetic diversity of the RV isolates, two distinct clades of RV were identified in 2004 [7], [8]. Furthermore, investigation of the molecular epidemiology of RV in southern China demonstrated that the long-distance migration, or transprovincial movement of dogs by humans from high-incidence regions may be one of the causes for the re-emergence of the disease [9]. Evolutionary dynamic analysis of RV based on the G gene [10] showed that the RV currently circulating in China is composed of three main groups and that the rabies viruses in China and Southeast Asia share a common ancestor [11].

Guangxi province is a severe epidemic region of rabies. The human cases of death due to rabies in Guangxi since 1997 rank the highest in China. More than 100 people have died of rabies each year since 2000, with a peak of 602 cases of death in 2004. Phylogenetic analysis based on the 3′-terminus of the N gene showed that RV isolates from Guangxi can be divided into four groups [12], although only two (I and II) are major causative factors of lethal rabies in humans and animals. In this study, we summarize the recent trends in the epidemiological characteristics, antigenicity, pathogenicity and phylogeny of street RV isolates that are highly prevalent in Guangxi.

## Materials and Methods

### Ethics statement

All animals experiments described in this paper were conducted according to the National Guideline on the Humane Treatment of Laboratory Animals Welfare (MOST of People's Republic of China, 2006) and approved by the Animal Welfare and the Animal Experimental Ethical Committee of Guangxi University (No. Xidakezi2000138). All husbandry procedures were conducted in compliance with the Animal Welfare Act and the Guide for the Care and Use of Laboratory Animals.

The sampling and collection protocol were approved by the Veterinary Administration of Guangxi. The brain samples of normal dogs and cats were collected and provided by the Guangxi Centre for Animal Disease Control and Prevention (the Animal CDC). The brain samples were collected into sterile plastic tubes. Mice used for viral isolation by the mouse inoculation test (MIT) were purchased from the Animal Centre of Guangxi Medical University. The mice were observed for 28 days post-injection, and then were euthanized in a container by halothane inhalant.

### Demographic investigation of rabies cases in Guangxi

For the surveillance of rabies in Guangxi, all counties reported the number of rabies cases in humans to the Guangxi Centre for Disease Control (CDC) and the Animal CDC, an Executive Department of the Guangxi Government. Most counties submitted data monthly, and all data were confirmed at the end of the year by the local CDC. The human rabies cases were diagnosed by histories of animal bites, scratches, or exposure to animal body fluids; and by clinical symptoms: hydrophobia or aerophobia, hypersalivation, excitation, and then paralytic signs in limbs, loss of incoordination, and tremors. Finally, most of the rabies cases were verified by indirect fluorescence assay (IFA).

### Sample collection, RT-PCR detection and virus isolation

From May 1999 to July 2010, 28 brain samples were obtained from rabid dogs, cattle, and pigs that were clinically suspected to have rabies; and 3040 brain samples, including 3032 normal dogs and 8 cats, were collected from different regions of Guangxi. All samples were provided by the Animal CDC as part of routine laboratory investigations for suspected cases. Samples were subjected to RT-PCR, and the positive samples were further used for RV isolation by MIT [12]. The mice were purchased from the Animal Centre of Guangxi Medical University. To comply with the “Animal Research: Reporting *In Vivo* Experiments” (ARRIVE) guidelines, all husbandry and experimental procedures were conducted in compliance with the Animal Welfare Act and the Guide for the Care and Use of Laboratory Animals.

Total viral RNA was extracted from brain samples using Trizol (Invitrogen, CA, U.S.) following the manufacturer's instructions. cDNA was synthesized using 2.5 µg total RNA, 1 µl (25 pMol/µL) sense primer, and 100 U MuMLV reverse transcriptase (Promega) in a 25 µl reaction volume. Each viral gene was amplified by PCR using ExTaq DNA polymerase.

### Virulence of RV isolates

The virulence of RV isolates was determined using four-week-old adult mice. The experiments were performed in microbiological safety cabinets in a biological safety laboratory (Group P2+). Dilution series of virus stock were prepared using DMEM in an ice bath. A total of 0.03 ml of each virus dilution was injected cerebrally into each adult mouse, using 4 mice per dilution. The mice were clinically observed for 28 days. Any death occurring within first 5 days was considered non-specific. The specific signs and symptoms of rabies were recorded as humane endpoints: ruffling of hair, loss of coordination, tremors, paralysis of fore or hind limbs, hind limb palsy, body weight loss, and convulsions. The mice with rabies clinical signs were euthanased. The total number of specific deaths for each dilution was calculated as the LD_50_ by the Reed-Muench method [13].

### Cell lines and cell culture

Neuro-2A (NA) cells (ATCC number: CCL 131) were grown in Dulbecco's Modified Eagle Medium (DMEM) containing 10% fetal calf serum. BHK-21 cells (ATCC number: CCL-10) were maintained in DMEM supplemented with 10% fetal calf serum.

### Antigenicity of RV isolates

The brains of original animals or adult mice infected with the street RV isolates were used to prepare sections. The sections were fixed with cold 30% acetone-70% methanol for 1 h and then stained with anti-G MAbs (kindly provided by Dr. Minamoto [14] of Gifu University, Japan) by IFA. The results were also verified in NA or BHK cells. FITC-conjugated goat antibody against mouse immunoglobulin was purchased from Sigma-Aldrich Corp (USA).

### Cloning and sequencing of RV G gene

The fragment of 1815 nucleotides containing complete G gene was amplified using a pair of primers GP1: 5′ ATC CCT CAA AAG ACT CAA GG 3′ (3293∼3314) and GP2: 5′ CCG TTA GTC ACT GAA ACT GC 3′ (5088∼5107) with cycling conditions of 95°C for 5 min; 35 cycles of 95°C for 1 min, 50°C for 1 min, and 72°C for 1 min. PCR products were purified and cloned into pMD18-T vector, and then sequenced by Takara Corp. Three clones were analyzed for each amplicon of each virus. Whenever clonal differences were identified, the other three clones were analyzed repeatedly until a consistent sequence was obtained. Sequence information was aligned and edited using the Vision X program.

### Phylogenetic analysis

The coding regions of the G gene in the genome dataset of the isolates (accession numbers in Table S1) were modeled for phylogenetic tree reconstruction as described previously [12]. Calculation of the homology of nucleotide sequences was carried out using genetic software (Windows version 6.0.1). Alignments of homologous sequences were performed with the Clustal method of the MegAlign program of the DNAStar version 7.1 package (DNAS_TAR_ Inc., U.S.). A neighbor-joining (NJ) tree for all DNA sequences was constructed using the Kimura 2-parameter model with MEGA4.0 software [15].

## Results

### Epidemiological characteristics of rabies in Guangxi

**Figure 1:**
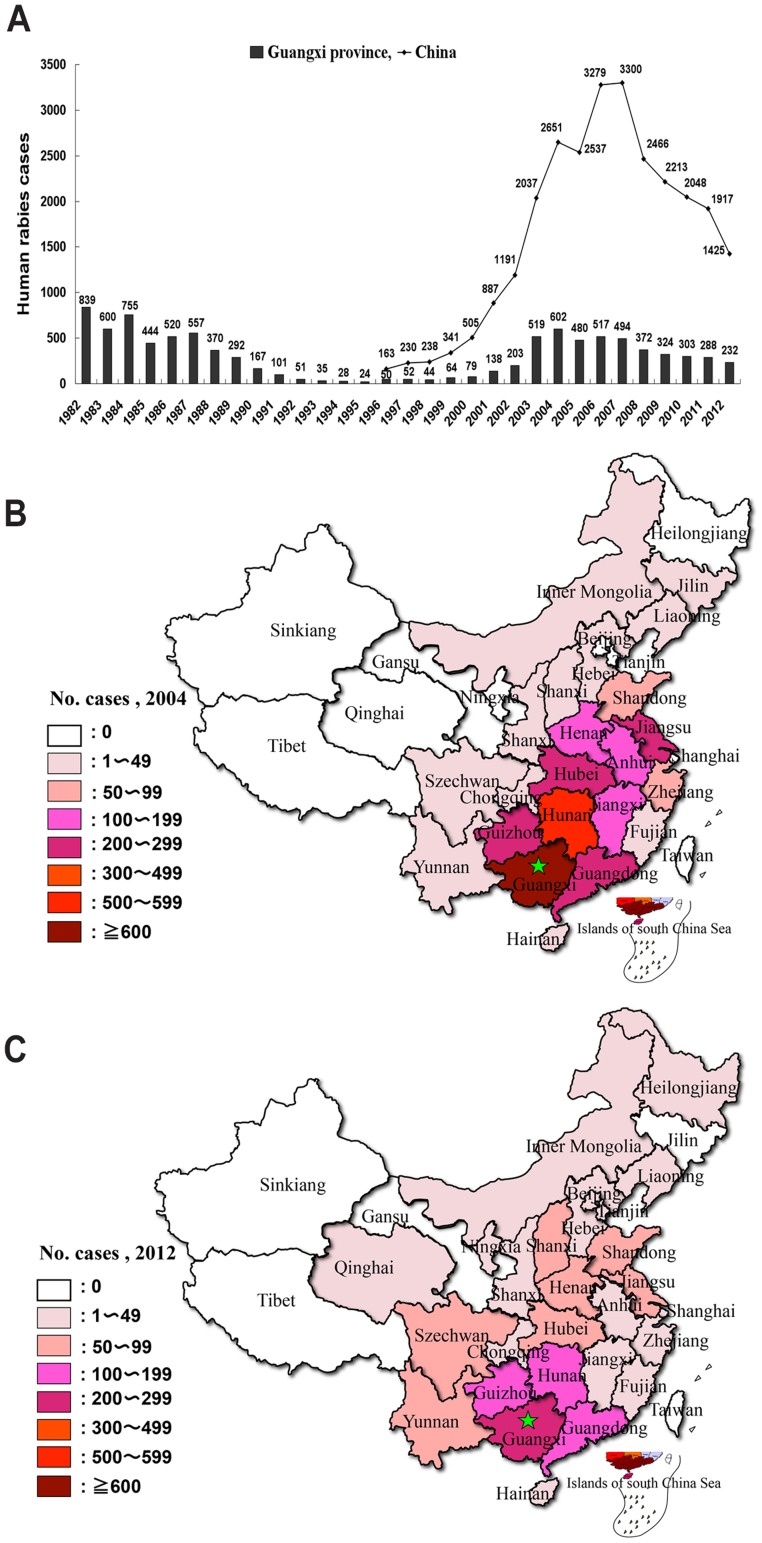
Epidemiology of rabies in Guangxi province of Southern China. (A) Numbers of recorded human rabies cases between 1982 and 2012 in Guangxi province and in all of China. (B) Distribution of human rabies cases in China in 2004. (C) Distribution of human rabies cases in China in 2012. The green star (★) indicates Guangxi province.

Since the first rabies outbreak during the 1950s and 1960s, Guangxi has undergone two re-emergences of rabies during the past three decades: one occurred in the 1980's, and we are currently in the midst of the second. The epidemiological characteristics of rabies in humans in Guangxi from 1982 to 2012 are shown in Figure 1A. The incidence of human rabies in Guangxi decreased from 839 in 1982 to 24 in 1995, but more than doubled to 50 cases the following year. Seven years later, in 2002, the human rabies cases increased sharply to 203, which is more than 8 times that of the incidence in 1995. In 2003, the incidence more than doubled again, with 519 cases of human rabies. A peak of 602 cases was observed in 2004, and has decreased gradually since then, but remains in the 200 s range. This geometric pattern of increased incidence over the ten years from 1995 to 2004 was observed in the whole of China; although the Guangxi province was the most serious epidemic region (Figure 1B and C).

### Detection and isolation of RV

**Figure 2:**
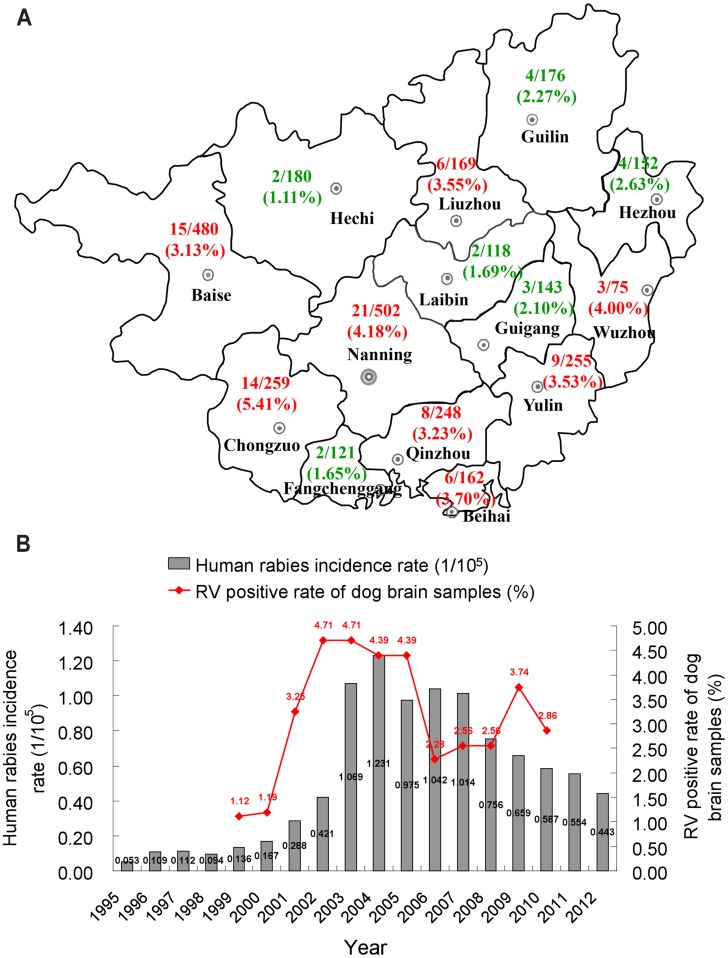
The relationship between human rabies incidence rate and RV positive rate of dog brain samples. (A) Distribution of the RV-positive dog brain sample. (B) The human rabies incidence rate is related with RV positive rate of dog brain samples.

To investigate the underlying cause of the RV outbreaks, 28 brain specimens were obtained from 20 dogs, six cattle and two pigs suspected of having rabies. The 28 brain specimens were verified to be RV positive by RT-PCR (100%), and 28 corresponding RV isolates were obtained by MIT. In addition, 3040 brain samples from 3032 normal dogs and 8 cats were collected from different areas of Guangxi from 1999 to 2010. 99 of the brain samples from normal dogs were determined to be RV-positive by RT-PCR. The RV positive rate in Chongzuo was highest, account for 5.41%; Nanning and Wuzhou were 4.18% and 4.0%, respectively. All districts with RV positive rate ≧3.0% including Beihai, Yulin, Qinzhou, Liuzhou and Baise locate in Guangxi (Fig. 2A). Of the 3040 samples, the RV positive rate was 3.26%, and the highest RV-positive rate was found during 2002–2005, with 59 of the 1301 brains from normal dogs collected from April 2002 to April 2005 being positive (4.39–4.71%) (Table 1).

**Table 1:** Rabies virus samples detected by RT-PCR and virus isolation.

Date	Rabid animals (dogs, cattle, pigs)		Normal (dogs and cats)		
	Number of samples	Virus isolation by MIT	Number of samples	Number of positive by RT-PCR (%)	Virus isolation by MIT
1999.3–2000.2	0	0	268	3 (1.12)	0
2000.3–2001.4	0	0	252	3 (1.19)	1
2002.4–2003.12	2	2	595	28 (4.71)	12
2004.1–2005.4	16	16	706	31 (4.39)	10
2006.2–2006.11	2	2	351	8 (2.28)	3
2007.2–2008.12	5	5	469	12 (2.56)	3
2009.1–2009.12	1	1	294	11 (3.74)	1
2010.1–2010.7	2	2	105	3 (2.86)	0
Total	28#	28	3040	99 (3.26*)	30

Note:

# , all samples from rabid animals were positive by RT-PCR;

*, average; MIT, mouse inoculation test.

A correlation between human rabies cases and detection of RV in animals was found: human rabies incidence rate increased following increased instances of RV positive rate of dog brain samples (Fig. 2B). For example, from 1999 to 2001, dog brains were 1.12–1.19% RV positive, and human rabies cases in Guangxi were 64, 79, and 138 (be equivalent to 0.136, 0.167 and 0.288 per 10^5^ population), respectively. However, from 2002 to 2005, RV positive brain samples increased to 4.71–4.39%, and the human rabies cases reached 203, 519, 602 and 480 (be equivalent to 0.421, 1.069, 1.231 and 0.975 per 10^5^ population), respectively for these years. Since 2006, the percentage of RV positive brain samples have declined to 2.28–2.86%, and the human rabies cases have decreased correspondingly.

### Virulence of the isolates

To determine the virulence of the isolated RV strains, six representative Guangxi isolates were tested using adult mice. The mice were cerebrally injected with diluted brain sample emulsions, 30 µl for adult mice (4 mice per group), and the clinical signs were scored post inoculation (Table 2).

**Table 2:** Pathogenicity determination for rabies virus isolates from Guangxi province.

Virus isolates	Animals injected	Genetic group	Origin	Date of collection	Incubation period (days)	Course of sickness (days)	Average death time (days)	LD_50_
GX01		I	Rabid dog	2004.03	5.33	2.33	7.5	10^−6.19^
GXHXB		I	Rabid dog	2007.04	5.0	2.0	7.0	10^−5.52^
GXLC9	Adult mice	II	Normal dog	2007.04	5.0	1.33	6.33	10^−5.85^
GX074		II	Normal dog	2003.02	5.33	1.33	6.67	10^−5.85^
GXNN2		II	Rabid dog	2007.08	4.0	4.5	7.5	10^−5.52^
GXPL		II	Rabid dog	2007.04	6.0	2.33	8.33	10^−5.35^

In adult mice, all isolates displayed a similar average incubation period of 4–6 d. The shortest incubation period was 4 d in mice infected with GXNN2. The course of sickness for the isolates also displayed a tight range from 1.33–4.5 d. The first signs of agitation appeared on 5 dpi, and the first paralytic signs of the hind limbs appeared at 6 dpi. All mice showed typical rabies signs: ruffled fur, lack of coordination, tremors, and paralysis of fore or hind limbs. The mice with typical rabies symptoms were euthanased. The 50% lethal dose (LD_50_) was calculated as 10^−5.35^/ml to 10^−6.19^/ml in adult mice (Table 2). These results suggest that all of the isolates tested caused a similar course of pathogenicity in mice.

### Antigenicity of RV

To evaluate the antigenicity of the RV isolates from Guangxi, 11 anti-G MAbs were used to determine the reactivity by IFA. Several representative isolates, including those of groups I and II, were selected for antigenicity testing. Among the anti-G MAbs, 8 showed strong reactivity with group I and II isolates, as well as the control strains RC-HL and ERA, with titers of ≥10000 (+++). However, the MAbs 12-14, 9-6, and 13-3 showed lower reactivity (100–1000 fold, +) with all isolates of group I and II, and also showed somewhat reduced reactivity with the control strains (Table 3). These results are suggestive of similar reactivity among the Guangxi isolates.

**Table 3:** Patterns of reactivity of MAbs with the G protein of rabies virus isolates from Guangxi*.

MAbs		Antigenic Site#	Guangxi isolates									Control strains
Name	Subclass of IgG		Group I			II						
			GX01	GXLA	GXHXB	GX074	GXNN2	GXLC9	GXPL	GXRSH01	RC-HL	ERA
15-13	G2b	I	+++	+++	+++	+++	+++	+++	+++	+++	+++	+++
15-10	G1	II-1	+++	+++	+++	+++	+++	+++	+++	+++	+++	+++
11-7	G2a	II-2	+++	+++	+++	+++	+++	+++	+++	+++	+++	+++
14-6	G2a	II-3	+++	+++	+++	+++	+++	+++	+++	+++	+++	+++
15-12	G2a	II-4	+++	+++	+++	+++	+++	+++	+++	+++	+++	+++
12-14	G2b	II-5	+	+	+	+	+	+	+	+	++	++
9-6	G2b	II-6	+	+	+	+	+	+	+	+	+	+
13-13	G2a	II-7	+++	+++	+++	+++	+++	+++	+++	+++	+++	+++
13-10	G2b	II-8	+++	++	+++	+++	+++	+++	+++	+++	+++	+++
13-3	G2b	II-9	+	+	+	+	+	+	+	+	+++	++
11-3	G2a	II-9,10	+++	+++	+++	+++	+++	+++	+++	+++	+++	+++

*Indirect fluorescent assay titer: +, 100–1,000; ++, 1000–10,000; +++, ≧10000.

# , The two antigenic sites (I and II) were defined on the G protein of the RC-HL virus by competitive binding assay using purified and biotinylated MAbs (reference 14).

### Sequencing and phylogenetic analysis based on the G gene

To investigate the origins of the Guangxi RV isolates, we selected 25 for sequencing and phylogenetic analysis, including representatives from a variety of districts and collection dates spanning the period from 2000 to 2007 (Table S2). The G gene was sequenced and aligned using ClustalW and subjected to phylogenetic tree reconstruction with the neighbor joining method [16].

Group I included 15 isolates with nucleotide homology of 97.6–99.9% and deduced amino acid homology of 98.1–100%. Group II included 10 isolates with nucleotide homology of 98.1–99.9% and deduced amino acid homology of 97.7–100%. However, between groups I and II, the homology for nucleotide and for deduced amino acid were 86.8–87.7% and 93.5–94.9%, respectively (Table S3). These results imply that group I obviously differs from the group II in evolution.

**Figure 3:**
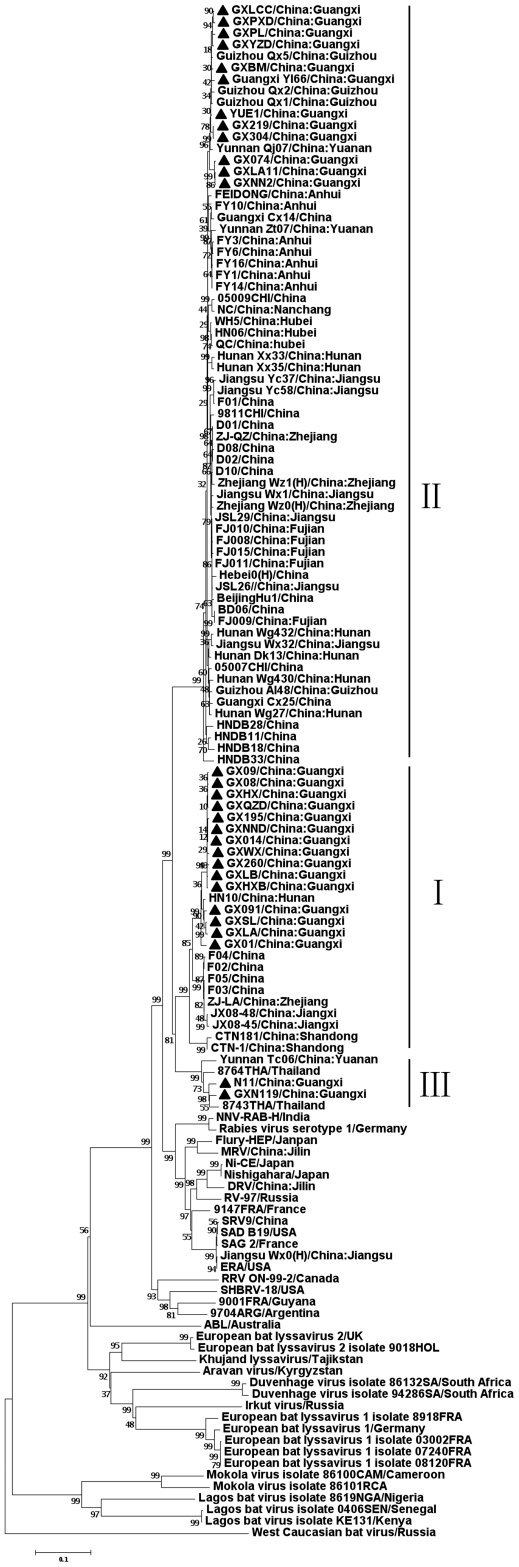
Phylogenetic tree of rabies viruses based on the G gene sequence. The rabies virus isolates from Guangxi are indicated by black triangles (▴).

A maximum likelihood phylogenetic tree was constructed for 133 complete G sequences (Figure 3). The Chinese isolates were independently divided into two major clusters, groups I and II, and were generally separate from the isolates of other countries. Isolates from the Guangxi, Hunan, and Guizhou provinces were of both groups I and II. It is noted that isolates from Guangxi exhibited similar topologies with strong bootstrap values in the two groups and were closely bonded. Group I contains isolates from Guangxi, Hunan, Guizhou, Fujian, Ningxia, Zhejiang and Jiangxi provinces, whilst group II contains isolates of from Guangxi, Hunan, Guizhou, Anhui, Jiangsu, Henan, and Yunnan province. The phylogeny indicated that the virus might be introduced from other provinces.

### Deduced amino acid variations based on the G gene

Comparison of the deduced amino acid sequence of the entire G gene of the isolates with that of the ERA strain, a vaccine for which is most commonly applied in Guangxi, showed several substitutions that distinguish the strains. Based on the G protein variations, our analyses revealed two specific substitutions, F-6V/I and V-7A, in the signal peptide for group I and three specific substitutions, L-4S, P-5S, and A-15V, for group II. However, the mature G protein was noted to contain 20 substitutions. Of which, four substitutions (A96S, L132F, N436S, and A447I) specific to group I, 13 substitutions (T90M, Y168C, S204G, T249I, P253S, S289T, V332I, Q382H, V427I, L474P, R463K Q486H, and T487N) specific to group II (Table 4). These results support the classification of the strains based on the nucleotide sequence.

**Table 4:** Specific mutational amino acids on glycoprotein of rabies virus isolates from Guangxi.

Strain	Group	The position of mutational amino acids																															
		−4	−5	−6	−7	−15	90	96	132	140	156	168	170	204	241	249	253	260	264	289	332	382	427	436	443	445	447	449	458	463	474	486	487
**ERA**		**L**	**P**	**F**	**V**	**A**	**T**	**A**	**L**	**A**	**S**	**Y**	**S**	**S**	**V**	**T**	**P**	**L**	**R**	**S**	**V**	**Q**	**V**	**N**	**L**	**A**	**A**	**T**	**M**	**R**	**L**	**Q**	**S**
GXLA				V	A			S	F		R								H					S	M		I						
GX08				I	A			S	F		R								H					S	M		I						
GX09				I	A			S	F		R								H					S	M		I						
GX014				I	A			S	F		R								H					S	M		I						
GX01				V	A			S	L		R								H					S	M		I						
GX091				V	A			S	F		R								H					S	M		I						
GX195	I			I	A			S	F		R								H					S	M		I						
GX260				I	A			S	F		R								H					S	M		I						
GXHX				I	A			S	F		R								H					S	M		I						
GXWX				I	A			S	F		R								H					S	M		I						
GXSL				V	A			S	F		R								H					S	M		I						
GXQZD				I	A			S	F		R								H					S	M		I						
GXHXB				I	A			S	F		R								H					S	M		I						
GXNND				I	A			S	F		R								H					S	M		I						
GXLB				I	A			S	F		R								H					S	M		I						
GX074		S	S			V	M				G	C		G		I	S		H	T	I	H	I		I					K	P	H	N
GXBM		S	S			V	M				G	C		G		I	S		H	T	I	H	I		I					K	P	H	N
GX219		S	S			V	M				G	C		G		I	S		H	T	I	H	I		I					K	P	H	N
GX304		S	S			V	M				G	C		G		I	S		H	T	I	H	I		I					K	P	H	N
GXPXD	II	S	S			V	M				G	C		G		I	S		H	T	I	H	I		I					K	P	H	N
GXLCC		S	S			V	M				G	C		G		I	S		H	T	I	H	I		I					K	P	H	N
GXPL		S	S			V	M				G	C		G		I			H	T	I	H	I		I					K	P	H	N
GXYZD		S	S			V		T			G	C		G		I	S		H	T	I	H	I		I					K	P	H	N
GX NN2		S	S			V	M				G	C		G		I	S		H	T	I	H	I		I					K	P	H	N
GXLA11		S	S			V	M				G			G		I	S		H	T	I	H	I		I					K	P	H	N

## Discussion

Guangxi is a severe epidemic region for rabies, with the highest rates in China. Over the past decade, 200–600 people in Guangxi have died of rabies each year (Fig. 1A). Domestic dogs are the principal vector, and 95% of human cases are associated with dog transmission. The major problems in controlling rabies are: 1) The understanding of rabies is extremely poor in rural areas; 2) People bitten by dogs often do not report the incidence and obtain treatment; 3) Dog populations in Guangxi are rising and currently estimated at more than 5 millions about 10% of the human population.

An outbreak of rabies in Guangxi occurred in the 1970s and reached a peak of 877 human cases in 1981. After the introduction of rabies vaccination for dogs, and increased efforts to eradicate stray dogs, the incidence of human rabies decreased to 24 cases in 1995. However, in 1996 the number of human rabies cases increased to 50, rose steeply to 203 in 2003, and reached a peak of 602 in 2004. A high rate of rabies incidence in Guangxi persists, although there has been a gradual decrease since 2004 (Fig. 1A). Nevertheless, it is clear that a re-emergence of rabies has occurred in Guangxi from 1996. However, the cases have been mainly limited to rural areas because pets in the cities tend to receive effective vaccination.

The rabies virus does not have carrier status [17]. However, there will be a degree of replication within the brains of animals at a pre-clinical stage of infection prior to the onset of clinical symptoms. These infected animals at a pre-clinical stage of infection probably secret RV via saliva and can transmit it to humans or other animals by biting. Thus, it is very important to understand whether or not the positive rate of the infected animals at a pre-clinical stage of infection relates to human cases of death. We collected 3040 brain samples from 3032 normal dogs and 8 cats from different areas of Guangxi between 1999 and 2010. Of these, 99 samples from normal dogs were determined to be RV-positive by RT-PCR, and found that human rabies cases increased correlated with RV in normal dogs (Fig. 2B). From the RV-positive samples, 30 RV isolates were obtained by MIT. Several isolates from rabid and normal dogs taken at different times showed similar pathogenicity in mice, indicating that the RV from Guangxi has stable virulence. It is noteworthy that the 99 positive samples were from normal dogs with no clinical symptoms, suggesting that normal dogs in Guangxi have a positivity rate of RV of 3.26%.

Over the past two decades, the use of MAbs for lyssavirus identification has significantly expanded the ability to differentiate individual viruses with reproducible results. We used 10 anti-N and 11 anti-G MAbs to assess the antigenicity of six representative RV isolates from Guangxi. The results showed that the isolates of group I/II that are mainly prevalent in Guangxi have similar reactivity patterns for the N protein (Data not shown). However, a small distinction of anti-G MAbs to the RV isolates occurred, in that the MAbs 12-14, 9-6 and 13-3 showed a weaker reactivity to the Guangxi isolates than the control RV strains. The reactivity patterns with anti-N/G MAbs suggests that the antigenicity of the Guangxi isolates is stable, but may contain some unique regional features.

Molecular typing to differentiate lyssavirus strains has been performed primarily on the N gene in order to evaluate concordance with former classifications by serotyping [18]. The N gene of RV is the most common target for genetic and adaptive evolution analysis because the gene is highly convergent [19]–[22]. For the Guangxi isolates, phylogenetic analysis of the N gene showed similar topologies among isolates with strong bootstrap values that were closely bonded. Alignment of the deduced amino acids revealed ten specific amino acid mutations on the N protein, coinciding with the phylogenetic analysis of the isolates (Data not shown).

Phylogenetic analysis and amino acid comparison demonstrated similar overall results for the RV isolates from Guangxi. The phylogenetic analysis confirms the findings of preliminary surveys, but also indicates that the major groupings can be explained by geographical parameters. Variations of the G protein demonstrated patterns similar to those obtained for the N protein (data not shown). Furthermore, RNA variations in the RV isolates from Guangxi are consistent with differences observed from other geographical regions. These finding suggest that there are strong geographical associations among RV isolates that might have contributed to the re-emergence of the disease in Guangxi.

Amino acid comparison demonstrated, those mutated amino acids in G protein do not specific to functional domains such as antigenic sites II (aa 34–42, and aa198–202) [23] and III (330–340) [24], MAbs sites (epitope sites 147, 184, 251, 263 and 264) [25]–[29], snake venom curareminetic neurotoxin (aa189–214) [30], and pathogenic sites (aa 37, 242, 255, 268 and 333) [31]–[33]. These results signified that the key amino acids in the functional domains were conserved.

Overall, a more precise and thorough documentation of confirmed rabies cases in humans and animals would give a better understanding of the epidemiological situation in the area. In view of above-mentioned reasons, people should maintain a high degree of vigilance when the stray dogs and cats approach.

## Supporting Information

Table S1 The rabies virus isolates available in this study (G gene).
(DOC)

Table S2 Origin of rabies virus isolates from Guangxi used in this study.
(DOC)

Table S3 Homologies of G gene of rabies isolates from Guangxi Province.
(DOC)
