# Supplementary material for: Re-emergence of Rabies in the Guangxi Province of Southern China
Source: PLoS Negl Trop Dis. 2014 Oct 2;8(10):e3114. doi: 10.1371/journal.pntd.0003114 (PMC4183421; doi:10.1371/journal.pntd.0003114)
Supplement: Table S1 — The rabies virus isolates available in this study (G gene). (DOC) [file pntd.0003114.s001.doc]

Table S1 The reference sequences of lyssaviruses G gene used

in this study

| Isolate | District | Host | GenBank accession number |
| --- | --- | --- | --- |
| Guizhou Qx1 | China Guizhou | Dog | EU267749 |
| Guizhou Qx2 | China Guizhou | Dog | EU267750 |
| Guizhou Qx5 | China Guizhou | Dog | EU267751 |
| Guangxi Yl66 | China | Dog | EU267744 |
| Yunnan Qj07 | China | Dog | EU275240 |
| BeijingHu1 | China | Homo sapiens | EU700029 |
| FJ010 | China Fujian | Dog | FJ866832 |
| Zhejiang Wz0(H) | China | Human | EF556198 |
| Hebei0(H) | China | Homo sapiens | EU267752 |
| FJ011 | China Fujian | Dog | FJ866833 |
| ZJ-QZ | China Zhejiang | Dog | FJ719758 |
| Zhejiang Wz1(H) | China | Homo sapiens | EU700030 |
| Hunan Xx35 | China | Dog | EU267771 |
| Jiangsu Wx1 | China | Dog | EU267773 |
| Hunan DK13 | China | Dog | EU267762 |
| Yunnan Zt07 | China | Dog | EU275241 |
| 05009CHI | China | Dog | EU086150 |
| HNDB28 | China | Dog | EU008927 |
| JX08-48 | China Jiangxi | Ferret badger | FJ719752 |
| ZJ-LA | China | Melogale moschata | FJ719756 |
| Jiangsu Wx0(H) | China | Homo sapiens | EU267772 |
| F01 | China | Chinese ferret badger | FJ825133 |
| D08 | China | Dog | FJ825129 |
| D10 | China | Dog | FJ825131 |
| JX08-47 | China Jiangxi | Ferret badger | FJ719749 |
| FY10 | China Anhui | Dog | DQ849053 |
| FY14 | China Anhui | Dog | DQ849056 |
| FY3 | China Anhui | Dog | DQ849046 |
| FJ015 | China Fujian | Dog | FJ866834 |
| HNDB33 | China | Dog | EU008928 |
| N11 | China Guangxi | Dog | DQ849069 |
| YUE1 | China Guangxi | Dog | DQ849070 |
| FY1 | China Anhui | Dog | DQ849044 |
| FY6 | China Anhui | Dog | DQ849049 |
| FY16 | China Anhui | Dog | DQ849058 |
| Guangxi Cx14 | China | Dog | EU267742 |
| FEIDONG | China Anhui | Dog | DQ849073 |
| HN06 | China Hubei | Dog | DQ849062 |
| QC | China Hubei | Homo sapiens | DQ849063 |
| WH5 | China Hubei | Dog | DQ849061 |
| NC | China Nanchang | Dog | DQ849064 |
| JSL26 | China Jiangsu | Dog | DQ849066 |
| JSL29 | China Jiangsu | Dog | DQ849068 |
| 9811CHI | China | Dog | EU086135 |
| Jiangsu Yc37 | China | Dog | EU267775 |
| Jiangsu Yc58 | China | Dog | EU267776 |
| Hunan Xx33 | China | Dog | EU267769 |
| Guangxi Cx25 | China | Dog | EU267743 |
| Hunan Wg27 | China | Dog | EU267766 |
| Guizhou Al48 | China | Dog | EU267748 |
| Hunan Wg430 | China | Dog | EU267767 |
| Jiangsu Wx32 | China | Dog | EU267774 |
| Hunan Wg432 | China | Dog | EU267768 |
| 05007CHI | China | Dog | EU086148 |
| HNDB11 | China | Dog | EU008924 |
| HNDB18 | China | Dog | EU008926 |
| F03 | China | Chinese ferret badger | FJ825134 |
| F05 | China | Chinese ferret badger | FJ825135 |
| Yunnan Tc06 | China | Dog | EU275242 |
| BD06 | China |  | EU549783 |
| FJ009 | China Fujian | Dog | FJ866836 |
| FJ008 | China Fujian | Dog | FJ866835 |
| D01 | China | Dog | FJ712193 |
| D02 | China | Dog | FJ712194 |
| HN10 | China Hunan |  | EU643590 |
| CTN181 | China Shandong | Vaccine strain | EF564174 |
| CTN-1 | China Shandong | Vaccine strain | FJ959397 |
| F04 | China | Chinese ferret badger | FJ712196 |
| F02 | China | Chinese ferret badger | FJ712195 |
| 8743THA | Thailand | Human | EU293121 |
| 8764THA | Thailand | Homo sapiens | EU293111 |
| Ni-CE | Japan | Lab strain | AB128149 |
| Nishigahara | Japan | Lab strain | AB044824 |
| RV-97 | Russia | Vaccine strain | EF542830 |
| DRV | China Jilin | Deer | DQ875051 |
| 9147FRA | France | Fox | EU293115 |
| SAD B19 | USA | Vaccine strain | M31046 |
| SAG 2 | France | Vaccine strain | EF206719 |
| SRV9 | China | Vaccine strain | AF499686 |
| ERA | USA | Vaccine/dog | EF206707 |
| HEP-Flury | Japan | Vaccine strain | AB085828 |
| MRV | China Henan | Mouse | DQ875050 |
| NNV-RAB-H | India | Homo sapiens | EF437215 |
| Rabies virus serotype 1 | Germany |  | AY956319 |
| 9704ARG | Argentina | Tadarida brasiliensis | EU293116 |
| SHBRV-18 | USA | Silver-haired bat | AY705373 |
| 9001FRA | Guyana | Dog | EU293113 |
| RRV ON-99-2 | Canada | Procyon lotor | EU311738 |
| Abl | Australia | Bat | AF418014 |
| European bat lyssavirus 1 isolate 07240FRA | France | Eptesicus serotinus | EU626552 |
| European bat lyssavirus 1 isolate 08120FRA | France | Eptesicus serotinus | EU626551 |
| European bat lyssavirus 1 isolate 03002FRA | France | Eptesicus serotinus | EU293109 |
| European bat lyssavirus 1 | Germany | Eptesicus serotinus | NC_009527 |
| European bat lyssavirus 1 isolate 8918FRA | France | Eptesicus serotinus | EU293112 |
| Duvenhage virus isolate 86132SA | South Africa | Homo sapiens | EU293119 |
| Duvenhage virus isolate 94286SA | South Africa | Miniopterus | EU293120 |
| Irkut virus | Russia | Murina leucogaster | EF614260 |
| European bat lyssavirus 2 isolate 9018HOL | Netherlands | Myotis dasycneme | EU293114 |
| European bat lyssavirus 2 | United Kingdom | Homo sapiens | NC_009528 |
| Khujand lyssavirus | Tajikistan |  | EF614261 |
| Aravan virus | Kyrgyzstan |  | EF614259 |
| Lagos bat virus isolate 0406SEN | Senegal | Eidolon helvum | EU293108 |
| Lagos bat virus isolate KE131 | Kenya | Eidolon helvum | EU259198 |
| Lagos bat virus isolate 8619NGA | Nigeria | Eidolon helvum | EU293110 |
| Mokola virus isolate 86100CAM | Cameroon | Shrew | EU293117 |
| Mokola virus isolate 86101RCA | Central African Republic | Rodent | EU293118 |
| West Caucasian bat virus | Russia | Miniopterus schreibersi | EF614258 |
