# Supplementary material for: Re-emergence of Rabies in the Guangxi Province of Southern China
Source: PLoS Negl Trop Dis. 2014 Oct 2;8(10):e3114. doi: 10.1371/journal.pntd.0003114 (PMC4183421; doi:10.1371/journal.pntd.0003114)
Supplement: Table S2 — Origin of rabies virus isolates from Guangxi used in this study. (DOC) [file pntd.0003114.s002.doc]

Table S2 Origin of rabies virus isolates from Guangxi used in this study

| Isolate | District | Host | Collection date | GenBank accession number |
| --- | --- | --- | --- | --- |
|  |  |  |  | G gene |
| GXN119 | Nanning | Dog | 2000.10 | GQ 472552 |
| GXLA | Nanning | Dog | 2003.01 | GQ 472549 |
| GX074 | Baise | Dog | 2003.02 | GQ 472539 |
| GX08 | Qinzhou | Dog | 2003.03 | GQ 472536 |
| GX09 | Qinzhou | Dog | 2003.03 | GQ 472537 |
| GXBM | Hechi | Dog | 2003.03 | GQ 472545 |
| GX014 | Chongzuo | Dog | 2003.04 | GQ 472538 |
| GX219 | Guigang | Dog | 2003.06 | GQ 472542 |
| GX304 | Fangchenggang | Dog | 2004.03 | GQ 472544 |
| GX01 | Guilin | Dog | 2004.03 | GQ 472535 |
| GX091 | Liuzhou | Dog | 2004.07 | GQ 472540 |
| GX195 | Wuzhou | Dog | 2004.10 | GQ 472541 |
| GX260 | Laibin | Dog | 2004.12 | GQ 472543 |
| GXHX | Hengxian | Dog | 2005.03 | GQ 472546 |
| GXSL | Shanglin | Cattle | 2005.03 | GQ 472558 |
| GXWX | Wuxuan | Pig | 2005.03 | GQ 472559 |
| GXPXD | Pinxiang | Dog | 2006.08 | GQ 472556 |
| GXQZD | Qinzhou | Dog | 2006.08 | GQ 472557 |
| GXHXB | Hengxian | Dog | 2007.04 | GQ 472547 |
| GXLCC | Liucheng | Dog | 2007.04 | GQ 472551 |
| GXPL | Panlong | Dog | 2007.04 | GQ 472555 |
| GXYZD | Yizhou | Dog | 2007.04 | GQ 472560 |
| GXNND | Nanning | Dog | 2007.04 | GQ 472554 |
| GXNN2 | Nanning | Dog | 2007.08 | GQ 472553 |
| GXLB | Liubei | Dog | 2007.08 | GQ 472550 |
| GXLA11 | Longan | Dog | 2007.11 | GQ 472548 |
