# Supplementary material for: Re-emergence of Rabies in the Guangxi Province of Southern China
Source: PLoS Negl Trop Dis. 2014 Oct 2;8(10):e3114. doi: 10.1371/journal.pntd.0003114 (PMC4183421; doi:10.1371/journal.pntd.0003114)
Supplement: Table S3 — Homologies of G gene of rabies isolates from Guangxi Province. (DOC) [file pntd.0003114.s003.doc]

Table S3 Homologies of G gene of rabies isolates from Guangxi Province

|  | 1 | 2 | 3 | 4 | 5 | 6 | 7 | 8 | 9 | 10 | 11 | 12 | 13 | 14 | 15 | 16 | 17 | 18 | 19 | 20 | 21 | 22 | 23 | 24 | 25 |  |  |
| --- | --- | --- | --- | --- | --- | --- | --- | --- | --- | --- | --- | --- | --- | --- | --- | --- | --- | --- | --- | --- | --- | --- | --- | --- | --- | --- | --- |
| 1 | *** | 98.5 | 98.7 | 98.7 | 98.1 | 98.9 | 98.5 | 98.7 | 98.7 | 98.1 | 99.0 | 98.7 | 98.5 | 98.7 | 98.5 | 93.7 | 93.9 | 93.5 | 93.5 | 93.7 | 93.9 | 93.7 | 93.7 | 93.9 | 93.9 | GXLA | Ⅰ |
| 2 | 97.8 | *** | 99.8 | 99.8 | 98.9 | 99.2 | 99.6 | 99.8 | 99.8 | 99.2 | 99.0 | 99.8 | 99.6 | 99.8 | 99.6 | 94.5 | 94.7 | 94.3 | 94.3 | 94.5 | 94.7 | 94.5 | 94.5 | 94.7 | 94.7 | GX08 |
| 3 | 97.8 | 99.9 | *** | 100 | 99.0 | 99.4 | 99.8 | 100 | 100 | 99.4 | 99.2 | 100 | 99.8 | 100 | 99.8 | 94.7 | 94.9 | 94.5 | 94.5 | 94.7 | 94.9 | 94.7 | 94.7 | 94.9 | 94.9 | GX09 |
| 4 | 97.9 | 99.9 | 99.9 | *** | 99.0 | 99.4 | 99.8 | 100 | 100 | 99.4 | 99.2 | 100 | 99.8 | 100 | 99.8 | 94.7 | 94.9 | 94.5 | 94.5 | 94.7 | 94.9 | 94.7 | 94.7 | 94.9 | 94.9 | GX014 |
| 5 | 98.1 | 98.0 | 98.0 | 98.2 | *** | 98.9 | 98.9 | 99.0 | 99.0 | 98.5 | 98.7 | 99.0 | 98.9 | 99.0 | 98.9 | 94.3 | 94.5 | 94.1 | 94.1 | 94.3 | 94.5 | 94.3 | 94.3 | 94.5 | 94.5 | GX01 |
| 6 | 98.8 | 98.1 | 98.1 | 98.2 | 98.3 | *** | 99.2 | 99.4 | 99.4 | 98.9 | 99.4 | 99.4 | 99.2 | 99.4 | 99.2 | 94.3 | 94.5 | 94.1 | 94.1 | 94.3 | 94.5 | 94.3 | 94.3 | 94.5 | 94.5 | GX091 |
| 7 | 97.7 | 99.6 | 99.6 | 99.7 | 97.9 | 98.0 | *** | 99.8 | 99.8 | 99.2 | 99.0 | 99.8 | 99.6 | 99.8 | 99.6 | 94.5 | 94.7 | 94.3 | 94.3 | 94.5 | 94.7 | 94.5 | 94.5 | 94.7 | 94.7 | GX195 |
| 8 | 97.6 | 99.6 | 99.6 | 99.7 | 97.8 | 97.9 | 99.4 | *** | 100 | 99.4 | 99.2 | 100 | 99.8 | 100 | 99.8 | 94.7 | 94.9 | 94.5 | 94.5 | 94.7 | 94.9 | 94.7 | 94.7 | 94.9 | 94.9 | GX260 |
| 9 | 97.8 | 99.9 | 99.9 | 99.9 | 98.1 | 98.2 | 99.7 | 99.6 | *** | 99.4 | 99.2 | 100 | 99.8 | 100 | 99.8 | 94.7 | 94.9 | 94.5 | 94.5 | 94.7 | 94.9 | 94.7 | 94.7 | 94.9 | 94.9 | GXHX |
| 10 | 97.6 | 99.5 | 99.5 | 99.6 | 97.8 | 97.8 | 99.4 | 99.4 | 99.6 | *** | 99.0 | 99.4 | 99.2 | 99.4 | 99.2 | 94.7 | 94.7 | 94.5 | 94.5 | 94.5 | 94.7 | 94.5 | 94.5 | 94.9 | 94.9 | GXWX |
| 11 | 99.5 | 98.0 | 98.0 | 98.2 | 98.4 | 99.0 | 97.9 | 97.8 | 98.1 | 97.9 | *** | 99.2 | 99.0 | 99.2 | 99.0 | 94.3 | 94.3 | 94.1 | 94.1 | 94.1 | 94.3 | 94.1 | 94.1 | 94.5 | 94.5 | GXSL |
| 12 | 97.7 | 99.7 | 99.7 | 99.7 | 97.9 | 98.0 | 99.5 | 99.4 | 99.8 | 99.4 | 97.9 | *** | 99.8 | 100 | 99.8 | 94.7 | 94.9 | 94.5 | 94.5 | 94.7 | 94.9 | 94.7 | 94.7 | 94.9 | 94.9 | GXQZD |
| 13 | 97.7 | 99.7 | 99.7 | 99.7 | 97.9 | 98.0 | 99.5 | 99.4 | 99.8 | 99.4 | 97.9 | 99.6 | *** | 99.8 | 99.6 | 94.5 | 94.7 | 94.3 | 94.3 | 94.5 | 94.7 | 94.5 | 94.5 | 94.7 | 94.7 | GXHXB |
| 14 | 97.8 | 99.8 | 99.8 | 99.9 | 98.1 | 98.2 | 99.7 | 99.6 | 99.9 | 99.6 | 98.1 | 99.7 | 99.7 | *** | 99.8 | 94.7 | 94.9 | 94.5 | 94.5 | 94.7 | 94.9 | 94.7 | 94.7 | 94.9 | 94.9 | GXNND |
| 15 | 97.8 | 99.7 | 99.7 | 99.9 | 98.0 | 98.1 | 99.6 | 99.6 | 99.8 | 99.5 | 98.0 | 99.6 | 99.6 | 99.8 | *** | 94.5 | 94.7 | 94.3 | 94.3 | 94.5 | 94.7 | 94.5 | 94.7 | 94.7 | 94.7 | GXLB |
| 16 | 87.4 | 87.2 | 87.2 | 87.4 | 87.7 | 87.8 | 87.1 | 87.1 | 87.3 | 87.2 | 87.7 | 87.1 | 87.3 | 87.3 | 87.4 | *** | 98.5 | 98.5 | 98.5 | 98.1 | 98.5 | 97.7 | 97.9 | 99.6 | 99.6 | GX074 | Ⅱ |
| 17 | 87.2 | 87.1 | 87.1 | 87.2 | 87.6 | 87.5 | 87.0 | 87.0 | 87.2 | 87.1 | 87.4 | 87.1 | 87.2 | 87.2 | 87.2 | 98.2 | *** | 99.2 | 99.2 | 99.2 | 99.6 | 98.9 | 99.0 | 98.5 | 98.5 | GXBM |
| 18 | 87.1 | 87.0 | 87.0 | 87.1 | 87.4 | 87.4 | 86.9 | 86.9 | 87.1 | 86.9 | 87.3 | 87.0 | 87.1 | 87.1 | 87.1 | 98.2 | 99.0 | *** | 99.6 | 98.9 | 99.2 | 98.5 | 98.7 | 98.5 | 98.5 | GX219 |
| 19 | 87.1 | 87.0 | 87.0 | 87.1 | 87.4 | 87.4 | 86.9 | 86.9 | 87.1 | 86.9 | 87.3 | 87.0 | 87.1 | 87.1 | 87.1 | 98.2 | 99.0 | 99.7 | *** | 98.9 | 99.2 | 98.5 | 98.7 | 98.5 | 98.5 | GX304 |
| 20 | 87.0 | 86.9 | 86.9 | 87.1 | 87.4 | 87.3 | 86.8 | 86.8 | 87.0 | 86.9 | 87.2 | 86.9 | 87.0 | 87.0 | 87.1 | 98.0 | 99.2 | 98.7 | 98.7 | *** | 99.2 | 98.5 | 98.7 | 98.1 | 98.1 | GXPXD |
| 21 | 87.1 | 87.1 | 87.1 | 87.2 | 87.5 | 87.4 | 86.9 | 86.9 | 87.1 | 87.0 | 87.4 | 87.1 | 87.1 | 87.1 | 87.2 | 98.2 | 99.4 | 98.9 | 98.9 | 99.7 | *** | 98.9 | 99.0 | 98.5 | 98.5 | GXLCC |
| 22 | 87.1 | 87.0 | 87.0 | 87.1 | 87.4 | 87.4 | 86.9 | 86.9 | 87.1 | 86.9 | 87.3 | 87.0 | 87.1 | 87.1 | 87.1 | 98.0 | 99.2 | 98.7 | 98.7 | 99.2 | 99.4 | *** | 98.3 | 97.7 | 97.7 | GXPL |
| 23 | 87.1 | 87.0 | 87.0 | 87.1 | 87.4 | 87.4 | 86.9 | 86.9 | 87.1 | 86.9 | 87.3 | 87.0 | 87.1 | 87.1 | 87.1 | 98.2 | 99.3 | 98.8 | 98.8 | 99.4 | 99.6 | 99.4 | *** | 97.9 | 97.9 | GXYZD |
| 24 | 87.3 | 87.2 | 87.2 | 87.4 | 87.7 | 87.6 | 87.1 | 87.1 | 87.3 | 87.2 | 87.6 | 87.1 | 87.3 | 87.3 | 87.4 | 99.7 | 98.3 | 98.2 | 98.2 | 98.1 | 98.3 | 98.1 | 98.2 | *** | 100 | GXNN2 |
| 25 | 87.4 | 87.3 | 87.3 | 87.4 | 87.8 | 87.7 | 87.2 | 87.2 | 87.4 | 87.2 | 87.6 | 87.2 | 87.4 | 87.4 | 87.4 | 99.7 | 98.2 | 98.2 | 98.2 | 98.0 | 98.2 | 98.0 | 98.2 | 99.9 | *** | GXLA11 |

The upper ﬁgures indicate the homologies of deduced amino acid sequences and the lower ﬁgures indicate the homologies of nucleotide sequences
